# Protein disulfide isomerase secretion following vascular injury initiates a regulatory pathway for thrombus formation

**DOI:** 10.1038/ncomms14151

**Published:** 2017-02-20

**Authors:** Sheryl R. Bowley, Chao Fang, Glenn Merrill-Skoloff, Barbara C. Furie, Bruce Furie

**Affiliations:** 1 Division of Hemostasis and Thrombosis, Beth Israel Deaconess Medical Center and Harvard Medical School, Boston, Massachusetts 02215, USA

## Abstract

Protein disulfide isomerase (PDI), secreted by platelets and endothelial cells on vascular injury, is required for thrombus formation. Using PDI variants that form mixed disulfide complexes with their substrates, we identify by kinetic trapping multiple substrate proteins, including vitronectin. Plasma vitronectin does not bind to αvβ3 or αIIbβ3 integrins on endothelial cells and platelets. The released PDI reduces disulfide bonds on plasma vitronectin, enabling vitronectin to bind to αVβ3 and αIIbβ3. *In vivo* studies of thrombus generation in mice demonstrate that vitronectin rapidly accumulates on the endothelium and the platelet thrombus following injury. This process requires PDI activity and promotes platelet accumulation and fibrin generation. We hypothesize that under physiologic conditions in the absence of secreted PDI, thrombus formation is suppressed and maintains a quiescent, patent vasculature. The release of PDI during vascular injury may serve as a regulatory switch that allows activation of proteins, among them vitronectin, critical for thrombus formation.

## Abstract (web summary)

What keeps blood from clotting in homeostasis is a puzzle. Here, the authors suggest that lack of the enzyme disulfide isomerase (PDI) in the blood is key, and show that PDI is secreted only after vascular injury to act on substrates that include vitronectin, affecting its binding to αVβ3 and αIIbβ3 integrins and enabling thrombus formation.

Thrombus formation is a host defence mechanism that protects the high-pressure mammalian circulatory system from significant loss of pressure following vascular injury. Limited proteolysis, cell activation and receptor conformational transitions are important mechanisms for rapidly transforming a quiescent system into an active system, as characterized by the activation of the blood coagulation cascade and fibrin generation, endothelial cell activation and degranulation and platelet thrombus formation^1^, but we have recently determined that protein disulfide isomerase (PDI) and several additional thiol isomerases may play an important role in maintaining the patency of the circulatory system. PDI and other thiol isomerases are required for thrombus formation and their absence from blood in the intact circulatory system is a mechanism to maintain patency in the absence of a breach. PDI is secreted from endothelial cells and bound platelets following vessel wall injury during thrombus formation^2^,^3^,^4^,^5^. From *in vivo* animal studies, PDI is required for both fibrin generation and platelet thrombus formation^2^,^3^. Similarly, ERp57 and ERp5, both members of the thiol isomerase family, also play a critical role in thrombus formation^6^,^7^,^8^,^9^. However, the molecular and cellular basis by which these thiol isomerases participate in the haemostatic process is unknown.

PDI, a prototype of a family of thiol isomerases with common thioredoxin-like domains and active sites characterized by the sequence CXXC (ref. 10), is an oxidoreductase involved in the formation of protein disulfide bonds during protein synthesis. PDI has two active thioredoxin domains, *a* and *a*′, each containing a CGHC motif, and two non-enzymatic domains, *b* and *b*′ potentially important for substrate recognition. We hypothesize that extracellular thiol isomerases are involved in critical modifications of vascular substrates during thrombus formation and propose that on vascular injury and the secretion of these enzymes, proteins critical to thrombus formation are converted from an inactive to an active form by changes in their disulfide bond structure. The modification of protein function by alteration of functional or allosteric disulfide bonds represents a novel and general regulatory mechanism^11^.

When the CGHC motif is reduced, a protein substrate disulfide can be reduced at the expense of the PDI active site. This reaction occurs through transient formation of a mixed disulfide between the N-terminal Cys in the PDI CGHC motif and a Cys in the substrate. Resolution of the mixed disulfide requires the second C-terminal Cys of the enzyme active site^12^,^13^. Mutation of the C-terminal Cys to Ala in the PDI active site leads to accumulation of the PDI-substrate mixed disulfide intermediate complex, allowing isolation and analysis of the target protein. This kinetic mechanism-based trapping of thiol isomerase substrates has been employed to examine the electron transport pathway in the endoplasmic reticulum^14^ and on cell surfaces^15^. We have adapted this procedure to study mixed disulfides involving PDI and its covalently bound substrates that originate in plasma and platelet releasate.

Vitronectin is among the protein substrates that formed mixed disulfide complexes with PDI. Vitronectin is present in plasma and platelets and binds to αVβ3 and αIIbβ3, plasminogen activator-1, the urokinase receptor, collagen and complement C5b-7 (ref. 16). This protein circulates as a single chain form that includes an N-terminal somatomedin B domain and a series of hemopexin-type domains. Vitronectin also circulates in a two chain disulfide-linked form following cleavage after Arg 379. Plasma vitronectin does not bind to αVβ3 (ref. 17). Studies of thrombus formation in live mice that lack vitronectin have revealed an role for vitronectin in thrombosis^18^,^19^, and we have confirmed the importance of this protein using vitronectin null mice in our model systems.

In the current study, we hypothesize that the absence of PDI from blood plays a critical role in maintaining patency of the circulatory system. On vascular injury, the release of PDI from the stimulated endothelium and bound platelets activates proteins, including vitronectin, critical for thrombus formation through modification of functional disulfide bonds^11^. PDI reduces either one or two disulfide bonds on plasma vitronectin that, we propose, enables this form of vitronectin to bind to β_3_ integrins and support thrombus formation.

## Results

### Kinetic trapping of protein substrates of extracellular PDI

**Figure 1:**
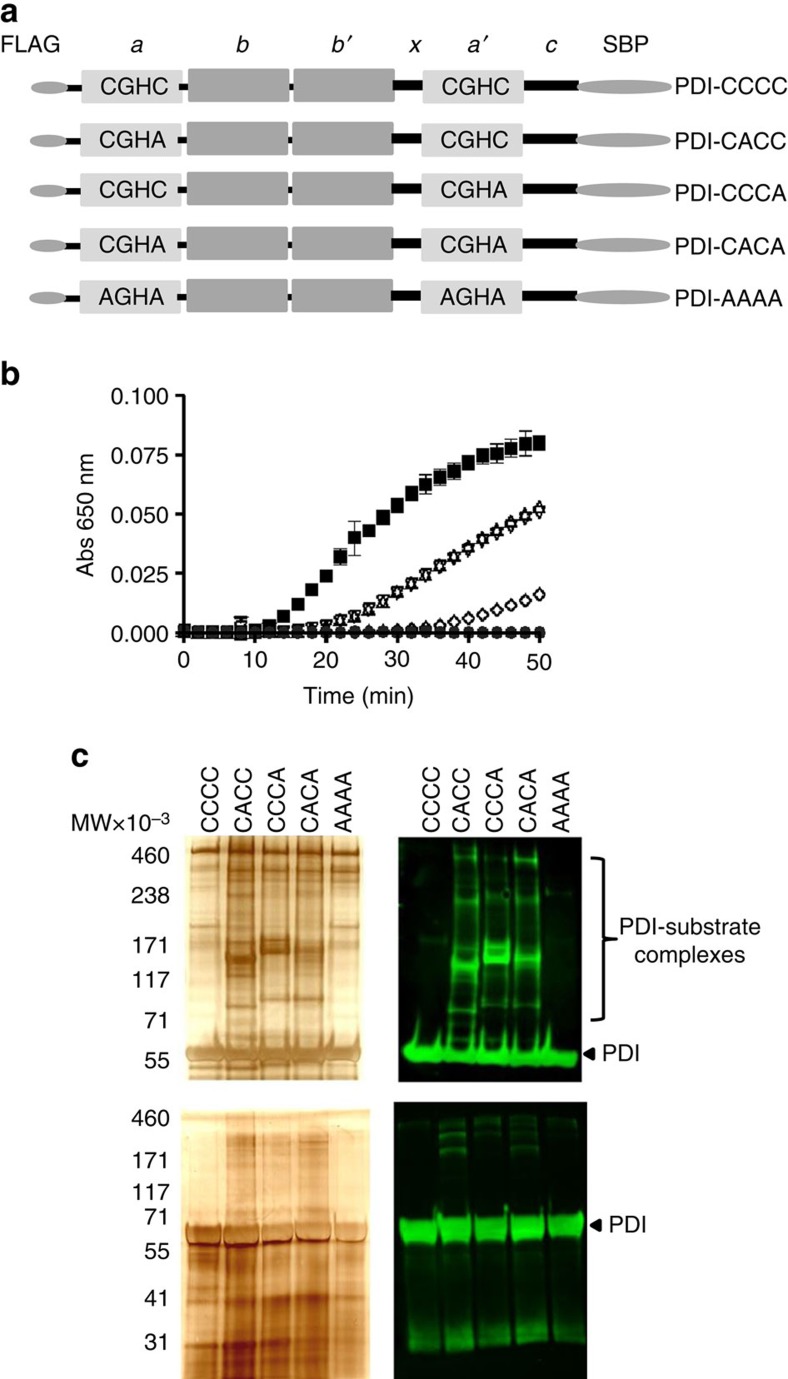
PDI and its variants for mechanism-based kinetic trapping of substrates. (**a**) Domains a and a′ (light grey) contain the catalytic motif (CGHC) whereas the b and b′ domains (dark grey) have no catalytic activity. ‘*x*' connects the *b*′ and *a*′ domains and there is a C-terminal extension c. CCCC, CACC, CCCA, CACA and AAAA indicate the amino acid residue in PDI at residues 53, 56, 397 and 400. FLAG is linked to the N-terminus and SBP (streptavidin binding peptide) to the C-terminus. (**b**) The reductase activities of PDI and the PDI variants were monitored by insulin reduction. PDI–CACC (inverted open triangle) and PDI–CCCA (open triangle) have impaired reductase activity compared with PDI–CCCC (black square) while PDI–CACA (open diamond) and PDI–AAAA (black circle) do not express reductase activity. The error bars represent standard deviation from the mean of 3 replicate experiments done in duplicate. (**c**) Kinetic trapping of PDI substrates was performed in platelet-rich plasma. PDI and PDI-associated proteins were isolated by streptavidin affinity chromatography. Samples were run under non-reducing conditions on 3–8 or 7% SDS–PAGE and proteins visualised by silver staining (Left) and by Western blot (Right) for PDI and PDI-substrate complexes (green), as blotted with fluorescently labelled anti-FLAG antibody. MW, molecular weight × 10^−3^. Upper panel, nonreduced; lower panel, reduced.

Kinetic trapping identified protein substrates of human PDI in plasma or secreted from platelets following cell stimulation. We generated three classes of recombinant PDI constructs: (1) wild type PDI, containing ^53^**C**GH**C**^56^ and ^397^**C**GH**C**^400^ located in the a domain and a′ domain respectively (PDI–CCCC); (2) three trapping variants in which an active site cysteine is mutated to alanine, precluding cleavage of the PDI-substrate complex: PDI–CACC: ^53^**C**GH**A**^56^ and ^397^**C**GH**C**^400^; PDI–CCCA: ^53^**C**GH**C**^56^ and ^397^**C**GH**A**^400^; PDI–CACA: ^53^**C**GH**A**^56^ and ^397^**C**GH**A**^400^; (3) inactive PDI in which cysteines were replaced by alanines: PDI–AAAA: ^53^**A**GH**A**^56^ and ^397^**A**GH**A**^400^ (Fig. 1a). These constructs were expressed with an N-terminal FLAG epitope and a C-terminal streptavidin binding peptide. Streptavidin affinity chromatography yielded PDI variants that migrated as a single band on SDS–polyacrylamide gel electrophoresis (SDS–PAGE). PDI variants PDI–CACC and PDI–CCCA have impaired reductase activity, whereas the PDI–CACA and PDI–AAAA variants have minimal or no reductase activity, respectively (Fig. 1b).

### PDI trapping mutants bind substrates in platelet-rich plasma

PDI and its variants were added to platelet-rich plasma containing Gly-Pro-Arg-Pro-NH_2_, an inhibitor of fibrin polymerization, before addition of collagen and thrombin. PDI and PDI-associated proteins from the resulting plasma and platelet releasate were affinity purified by streptavidin then subjected to SDS–PAGE. Wild type PDI–CCCC and the inactive PDI variant (PDI–AAAA) showed comparable banding patterns by silver stain but no fluorescence staining upon Western blotting for PDI except at the position of uncomplexed PDI (Fig. 1c, upper panel). The reaction of the three trapping mutants with the plasma samples produced mixed disulfide complexes as visualised on non-reduced SDS–PAGE and the corresponding Western blot probed for complexes containing PDI (Fig. 1c, upper panel). On addition of DTT, these PDI complexes disappeared, confirming the role of disulfide bonds in complex formation (Fig. 1c, lower panel).

### 2D electrophoresis of PDI-substrate complexes

**Figure 2:**
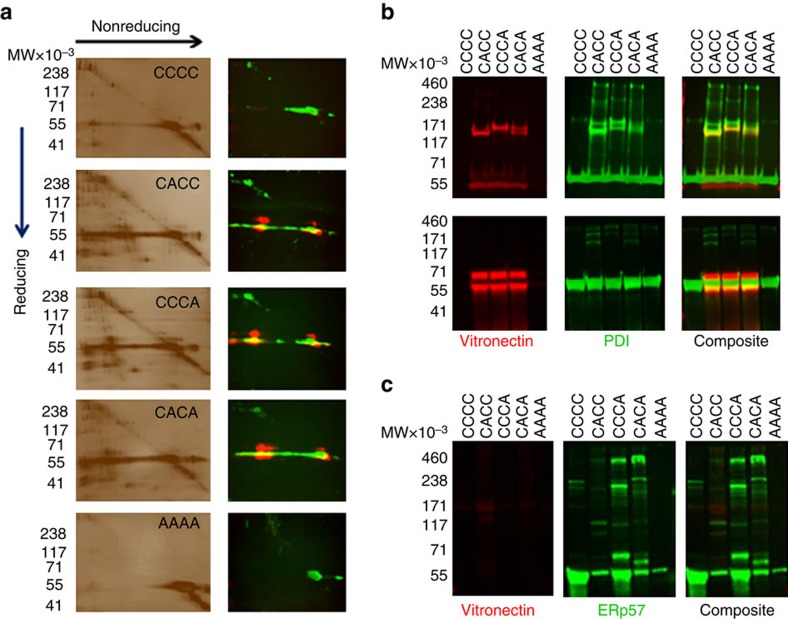
Western blot of trapped PDI substrates. (**a**) 2D electrophoresis of PDI and variants complexed to substrates from platelet-rich plasma. PDI complexes were isolated and analysed by 2D SDS–PAGE and Western blotting. Samples were run under nonreducing conditions, the gel treated with DTT, newly formed thiols alkylated with NEM, and the proteins in the gel strip run in the second dimension. Left: silver stain. Right: Western blot: PDI (green) using anti-FLAG antibody and vitronectin (red) using anti-vitronectin antibody. (**b**) Kinetic trapping with PDI and PDI variants in platelet-rich plasma. Samples were analysed by SDS–PAGE, blotted for vitronectin (red) and PDI (green) and detected by immunofluorescence as in A. (**c**) Kinetic trapping experiments with ERp57 and ERp57 variants were performed in parallel in platelet-rich plasma. ERp57 (green) and vitronectin (red) were detected by immunofluorescence as in A. MW, molecular weight × 10^−3^.

To identify protein substrates of PDI via its reductase activity, trapping experiment was performed in platelet rich plasma as before and resulting PDI complexes were analysed by 2D SDS–PAGE (Fig. 2a). Samples were run under nonreducing conditions in the first dimension. After in-gel reduction with DTT and alkylation with NEM, the proteins were separated by SDS–PAGE in the second dimension. This analysis distinguishes between proteins disulfide-linked to PDI and those bound noncovalently to PDI. Disulfide-linked PDI complexes on reduction separated into individual components that appear on the gel (Fig. 2a). These proteins include the ‘trapped' substrates and monomeric PDI. In contrast to PDI–CCCC and PDI–AAAA, all three trapping PDI variants highlight similar proteins off of the diagonal. The protein complexes trapped with PDI–CACA (Fig. 2a) were excised and identified by mass spectrometry. Proteins included vitronectin, complement factor 3, complement factor 5, C4b-binding protein, α2-macroglobulin, protein S, histidine-rich glycoprotein, thrombospondin 1, prothrombin and CD5 antigen-like protein. From this list, we independently verified by Western blotting that vitronectin, histidine-rich glycoprotein, protein S and prothrombin are redox substrates of PDI.

To confirm vitronectin as a PDI substrate, the 2D gels analysing the mixed disulfide complexes were probed with anti-vitronectin antibody (Fig. 2a, right). Reduced forms of vitronectin, with molecular weights of about 60,000 and 65,000, were bound to the trapping variants. Some vitronectin forms of approximately the same molecular weight are visible on the diagonal.

### Vitronectin is a substrate of PDI but not of ERp57

PDI–CACC, PDI–CCCA and PDI–CACA formed mixed disulfide complexes with vitronectin when added to platelet-rich plasma (Fig. 2b). Neither the PDI–CCCC nor the inactive PDI-AAAA formed mixed disulfide complexes with vitronectin. PDI nor its variants were detected using anti-FLAG antibodies whereas vitronectin was detected using an anti-vitronectin antibody. Secondary antibodies labelled with Alexa 647 and Alexa 488 allowed simultaneous detection of PDI and vitronectin in non-reduced (Fig. 2b, upper) and reduced (Fig. 2b, lower) SDS gels. The PDI trapping variants reacted with vitronectin in platelet-rich plasma to form a mixed disulfide complex of PDI and vitronectin with a molecular weight of approximately 120,000 (Fig. 2b, upper). On reduction, two forms of vitronectin are visualised. Since all PDI trapping mutants reacted with vitronectin, the catalytic active site in the a or a' domains is capable of reducing disulfide bonds of vitronectin.

Although there are two potential trapping sites in PDI–CACA, there is no band in the unreduced 2D SDS–PAGE for this variant, consistent with a complex of two vitronectin molecules linked to PDI. Instead, the PDI–CACA mutant showed a doublet, with each band corresponding to the individual band seen in the PDI–CACC and PDI–CCCA complex. This indicates a 1:1 stoichiometry between PDI and vitronectin. Vitronectin contains thrombin cleavage sites at Arg^370^-Asn^371^ and Arg^305^-Thr^306^ (ref. 20). Since thrombin was exogenously added during the trapping experiments to mimic conditions during thrombus formation, proteolysis yielded vitronectin species of 63,000 and 58,000.

ERp57 has a similar size and domain structure as PDI with 33% identity in their amino acid sequences. Both enzymes have catalytic *a* and *a′* domains sharing 50% amino acid identity and the CGHC motif^21^. To determine whether PDI and ERp57 share substrate specificity, we performed kinetic trapping experiments in platelet-rich plasma with ERp57 using ERp57-CCCC, ERp57-CACC, ERp57-CCCA, ERp57-CACA, and ERp57-AAAA. Analysis of the experiment by SDS–PAGE and Western blot showed trapping variants of ERp57 produce a fingerprint of mixed disulfide complexes distinct from PDI, suggesting unique sets of substrates (Fig. 2c). Immunoblot for the presence of vitronectin among the ERp57 mixed disulfide complexes indicate that none of the ERp57 trapping variants produce a disulfide-linked complex containing vitronectin. This demonstration of substrate specificity distinguishes PDI and ERp57.

**Figure 3:**
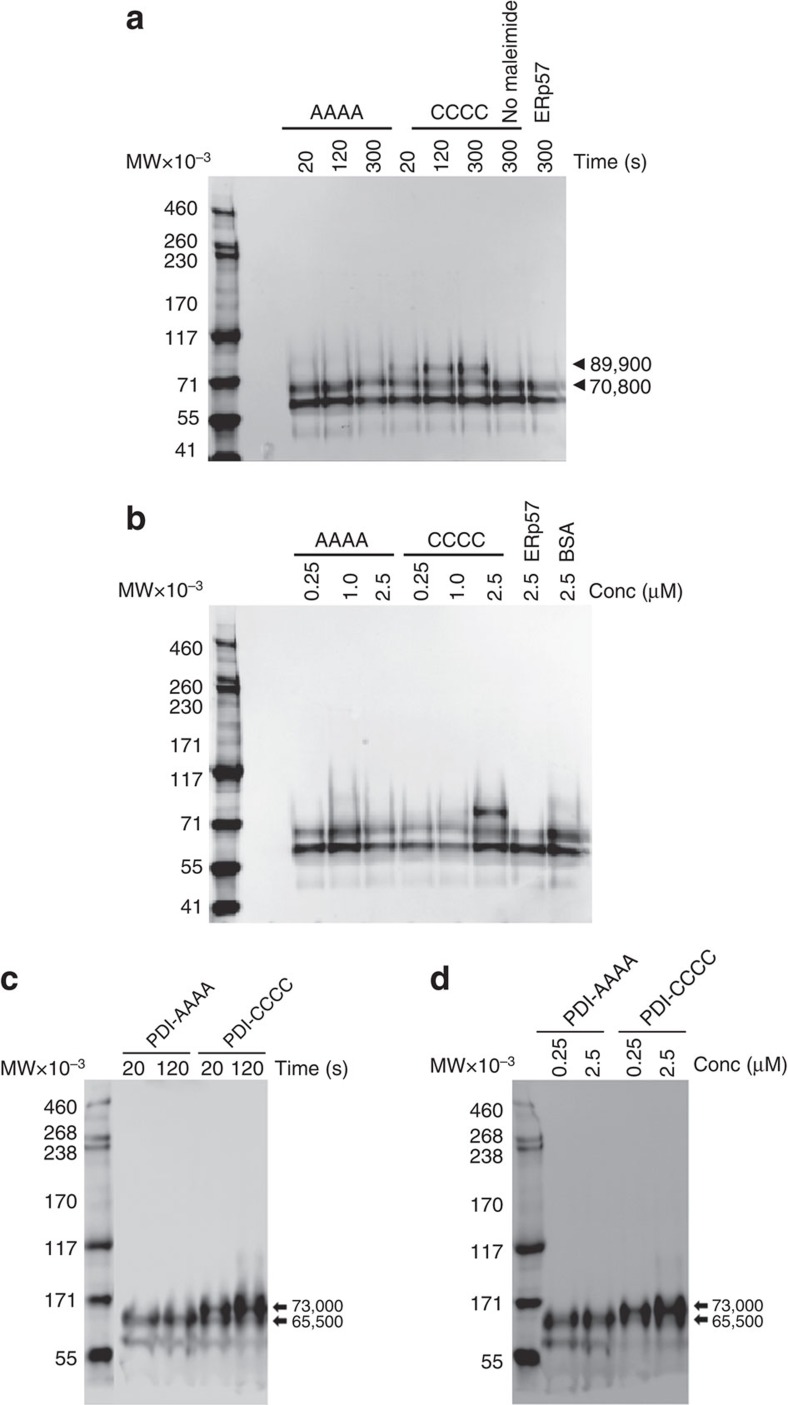
PDI reductase activity cleaves two disulfide bounds on vitronectin. PDI-CCCC, ERp57-CCCC or PDI–AAAA was added to platelet-poor plasma and disulfide reduction in vitronectin monitored by alkylation of new sulhydryls with 5,000 MW PEG-maleimide. Proteins were separated by SDS–PAGE on 3–8% gels and vitronectin probed by Western blotting. (**a**) Time dependence, from 0 to 5 min at 1 μM enzyme; (**b**) Dose dependence, 0.25 to 2.5 μM for 5 min. As above except alkylation of new sulhydryls was performed with 2,000 MW PEG-maleimide. (**c**) Time dependence, from 20 to 120 s at 1 μM enzyme; (**d**) Dose dependence, 0.25 to 2.5 μM for 5 min.

To further characterize the reductase reaction between PDI and plasma vitronectin, we determined the number of disulfide bonds in vitronectin that are reduced by PDI. Since purified plasma vitronectin assumes different conformations depending on the method of purification (that is, multimer formation with urea treatment and conformational changes after heparin-affinity purification^22^,^23^), we performed the PDI-vitronectin reaction using vitronectin in fresh platelet-poor plasma. Since vitronectin has two free thiols, the plasma was initially treated with NEM and excess NEM removed by dialysis, then reacted with either PDI–CCCC or PDI–AAAA. The reduction of disulfide bonds and subsequent alkylation of the new thiols in vitronectin were visualised by immunoblotting and monitoring gel mobility shifts associated with the incorporation of PEG_5000_-maleimide (Fig. 3). The time-dependent (Fig. 3a) and dose-dependent (Fig. 3b) reduction of plasma vitronectin by PDI–CCCC but not PDI–AAAA led to the appearance of vitronectin species of increased molecular weight at 89,900. This molecular weight increase corresponds to the incorporation of 3.6±0.06 moles of the PEG_5000_-maleimide per mole of vitronectin. No incorporation of PEG_5000_-maleimide was observed when ERp57 was added to plasma. Similar results, with the appropriate increases in the molecular weights, were obtained when PEG_2000_-maleimide was employed (Fig. 3c,d). These findings are consistent with the reduction of two disulfide bonds in vitronectin.

### PDI reduces disulfide bonds in vitronectin

**Figure 4:**
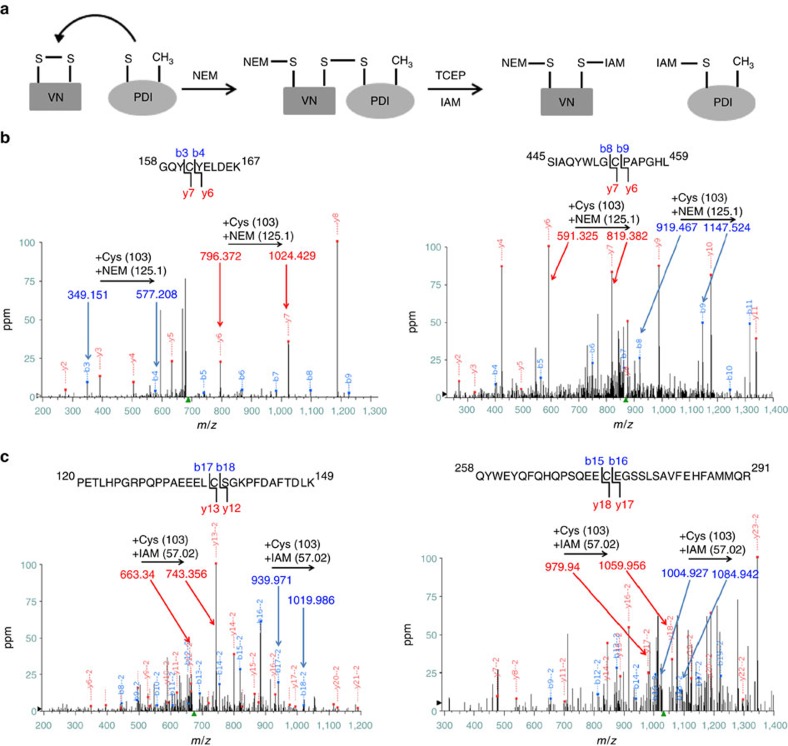
PDI cleaves Cys137-Cys161 and Cys 274–453 disulfide bonds in the hemopexin-like domains of vitronectin. (**a**) The mixed disulfide complex between PDI and vitronectin followed by thiol alkylation with NEM and IAM identified PDI-cleaved vitronectin disulfide bonds by mass spectrometry. (**b**) The tryptic sequence containing 158–167 was observed as a singly-charged species (left). The mass difference between b_3_ and b_4_ ions shows that Cys 161 is alkylated with NEM. A singly-charged peptide containing 445–453 shows a mass increase between *b*_8_ and *b*_9_ ions consistent with NEM-alkylated Cys 453 (right). (**c**) Cys 137 is alkylated with IAM from the doubly charged peptide 120–149 (left). The carbamidomethyl cysteine is evident from the mass difference of b_17_ and b_18_ ions. The mass difference between b_15_ and b_16_ ions from the doubly-charged peptide containing 258–291 is consistent with carbamidomethylation at Cys 274 (right).

To identify the disulfide bonds in vitronectin that are cleaved by PDI a trapping reaction was performed with PDI-CACA in platelet-free plasma and the mixed disulfide complex between PDI and vitronectin isolated followed by alkylation with NEM then reduction and alkylation with iodoacetamide. The initial alkylation step with NEM derivatized the free sulfyhydryls present in plasma vitronectin as well as the newly formed thiol(s) resulting from cleavage of disulfide bonds by PDI (Fig. 4a). Hence, a disulfide bond in vitronectin that was cleaved by PDI can be identified by the presence of a Cys-NEM. Figure 4b shows the fragmentation ions from the peptide GQYCYELDEK (residues 158–167) in which the difference between *b*_3_ and *b*_4_ ions as well as *y*_6_ and *y*_7_ ions indicates NEM alkylation at Cys 161. A second NEM modification was observed in the peptide SIAQYWLGCPAPGHL (residues 445–459) (Fig. 4b) in which the mass increase between *b*_8_ and *b*_9_ ions and the complementary *y*_6_ and *y*_7_ ions corresponds to NEM alkylation at Cys 453. The cysteines paired to Cys 161 and Cys 453 (Cys 137 and Cys 274, respectively) showed carbamidomethylation with iodoacetomide (Fig. 4c). These results indicate that PDI cleaves the disulfide bonds Cys 137–Cys 161 and Cys 274– Cys 453 in the hemopexin-like domains of plasma vitronectin. While Cys 137–Cys 161 is an intradomain linkage^24^, Cys 274–Cys 453 is an interdomain bridge that links the C-terminal fragment to the central part of the molecule^25^.

### Reduction of disulfides correlates with β3 binding

**Figure 5:**
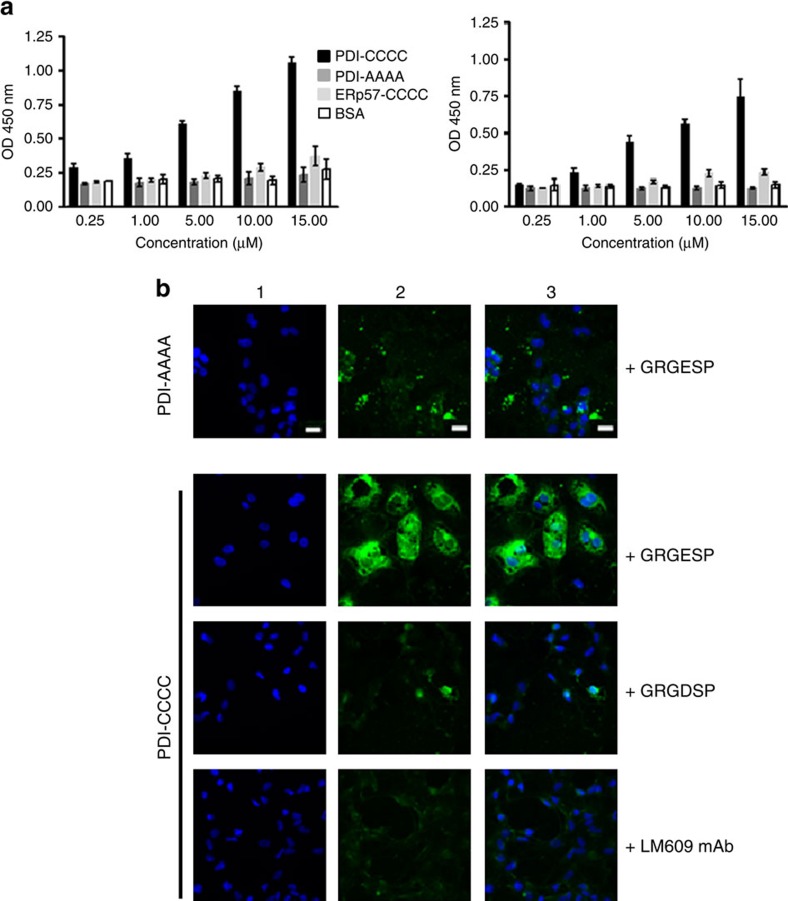
PDI enables binding to β_3_ integrins via the RGD motif. (**a**) Platelet-free plasma was treated with PDI-CCCC, PDI-AAAA, ERp57-CCCC or BSA, and the reaction terminated with NEM. The treated plasma was added to immobilized α_V_β_3_ or α_IIb_β_3_. After washing, bound vitronectin was quantitated with anti-vitronectin. PDI-CCCC, black; PDI-AAAA, dark grey; ERp57-CCCC, light grey; BSA, white. Left, α_V_β_3_; Right, α_IIb_β_3_. The error bars represent standard deviation from the mean of 2 replicate experiments done in triplicate. (**b**) Vitronectin from plasma treated with PDI-CCCC binds to activated HUVECs in the presence of G**RGE**SP whereas vitronectin from plasma treated with PDI-AAAA showed minimal binding. Binding of vitronectin from plasma treated with PDI-CCCC to activated cells pre-treated with G**RGE**SP,G**RGD**SP, or α_V_β_3_-function blocking LM609 antibody. Scale bar (white): 15 μm. Lane 1, DAPI; Lane 2, vitronectin; Lane 3, merge.

Native plasma vitronectin does not bind to integrin adhesion receptors and is not an adhesive glycoprotein^17^. We hypothesized that PDI-catalysed disulfide bond reduction in plasma vitronectin allows for binding to its receptors, α_V_β_3_ and α_IIb_β_3_. We evaluated the functional effect of vitronectin reduction by assessing vitronectin binding to immobilized α_V_β_3_ integrin. Binding of wild type PDI-treated plasma vitronectin to α_V_β_3_ integrin was increased as a function of PDI concentration (Fig. 5a, left). Up to 4-fold increase in bound vitronectin was observed at the highest PDI concentration tested (15 μM). In contrast, plasma treated with increasing concentrations of inactive PDI-AAAA showed vitronectin-α_V_β_3_ integrin binding similar to background binding. Wild type ERp57-CCCC did not increase vitronectin binding, consistent with our finding that ERp57-CCCC does not interact with vitronectin.

Using the same experimental approach we determined whether PDI-reduced plasma vitronectin also binds to α_IIb_β_3_ in this solid-phase binding assay. Increasing concentrations of PDI showed in excess of three-fold increase in bound vitronectin at the highest PDI concentration studied (Fig. 5a, right). These results demonstrate that PDI-catalysed reduction of a disulfide bond in vitronectin also enables its binding to α_IIb_β_3_. This suggests that the β3 subunit is the dominant ligand for the reduced form of vitronectin.

### Binding of vitronectin to β3 receptors on HUVECs

The binding of PDI-reduced vitronectin to β_3_ was confirmed by cellular assay using cultured HUVECs. Cells were activated with thrombin and Mn^2+^. Because HUVECs secrete endogenous PDI upon activation, the endothelial cell releasate was discarded and the cells were washed before the addition of plasma treated with either wild type PDI-CCCC or inactive PDI-AAAA. The amount of vitronectin bound to the cells was visualised by immunofluorescence microscopy using anti-vitronectin antibody. In the presence of the peptide Arg-Gly-Glu (RGE), minimal vitronectin binding to HUVECs was detected when plasma was treated with the inactive PDI-AAAA whereas vitronectin staining was markedly increased in PDI–CCCC-treated plasma (Fig. 5b). Pretreatment of cells with a peptide containing the sequence Arg-Gly-Asp (RGD) inhibited binding of vitronectin from the wild type PDI-treated plasma. Furthermore, the vitronectin binding observed in PDI-CCCC-treated plasma was abolished by pretreatment of cells with LM609, a function-blocking monoclonal antibody to the α_V_β_3_ complex. This indicates that the interaction occurs specifically through α_V_β_3_ integrin. PDI–AAAA failed to induce binding of vitronectin to HUVECs. These results suggest that active PDI cleaves a disulfide bond in vitronectin that exposes the RGD site, enabling its binding to α_V_β_3_.

### Vitronectin accumulates after vascular injury *in vivo*

**Figure 6:**
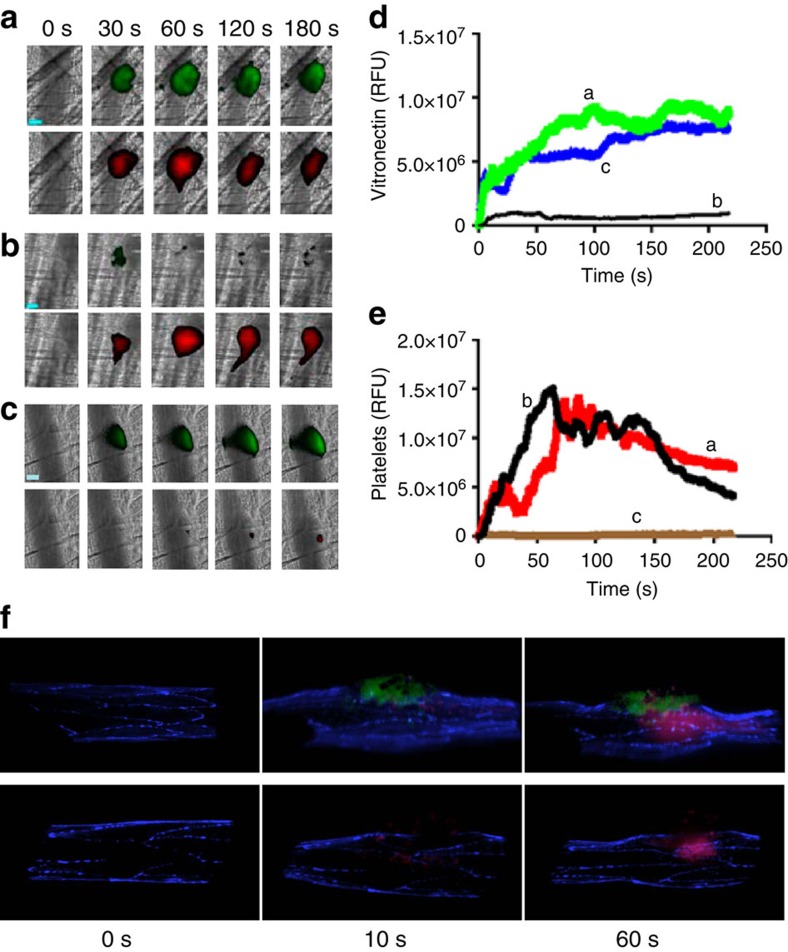
Vitronectin accumulates at the injury site during thrombus formation *in vivo*. Images of vitronectin (green) and platelets (red) were monitored by intravital microscopy following laser-induced vessel wall injury using platelet-specific anti-CD42b antibody conjugated to Dylight 649 (0.1 μg g^−1^ body weight) and rat anti-mouse vitronectin monoclonal antibody conjugated to Dylight 488 (0.5 μg g^−1^ body weight) infused into a mouse 5–10 min before injury. (**a**) Images depicting platelets (red) and vitronectin (green) were monitored following injury. (**b**) Images depicting platelets (red) and an irrelevant isotype-matched antibody (green) were monitored following injury. (**c**) Eptifibatide (10 μg g^−1^ mouse) was infused, and reinfused every 20 min. Images depicting platelets (red) and vitronectin (green) were monitored following injury. (**d**) Kinetics of vitronectin binding to the endothelium and the developing thrombus. Median integrated fluorescence from at least 28 thrombi in each group is shown in the presence of (**a**) anti-vitronectin and anti-platelet antibodies; (**b**) control irrelevant antibody of the same isotype as anti-vitronectin and anti-platelet antibodies; (**c**) anti-vitronectin antibody and anti-platelet antibodies following the infusion of eptifibatide. (**e**) As in D the kinetics of platelet accumulation is shown. Scale bar (cyan): 20 μm. (**f**) Confocal intravital reconstructions of a thrombus following vessel wall injury: The kinetics of the appearance of vitronectin and platelets within the context of the vessel wall are shown before vessel wall injury (Time 0), at 10 s following vessel wall injury (Time 10 s) and at 60 s following vessel wall injury (Time 60 s). In the upper panel, rat anti-mouse vitronectin monoclonal antibody, anti-CD42b antibody conjugated to Dylight 649 (0.1 μg g^−1^ body weight), and anti-CD31 antibody conjugated to Alexa 561 (1 μg g^−1^ body weight) were infused into a mouse 5 min before injury. These antibodies detected vitronectin (green), platelets (red) and PECAM (bue) respectively. In the lower panel, an isotype control IgG conjugated to Dylight 488 (1 μg g^−1^ body weight) (green) was substituted for the anti-vitronectin antibody.

Plasma vitronectin does not interact with αVβ3 (ref. 17). Using intravital microscopy and anti-vitronectin antibody conjugated to Dylight488 to detect bound vitronectin in a living mouse, we confirmed that vitronectin could not be detected on the unperturbed arteriolar wall in the cremaster microvasculature before vessel injury. To image the accumulation of vitronectin in the developing thrombus after laser-induced injury in an arteriole, vitronectin was visualised with the same antibody and platelet thrombus formation was visualised using anti-CD42b conjugated to Dylight 649. Vitronectin immediately accumulated following injury and was localised to the injury site (Fig. 6a,d). The kinetics of the median integrated fluorescence associated with vitronectin showed the very early appearance of vitronectin during thrombus formation—before platelet accumulation, suggesting vitronectin binding to the endothelium at the injury site (Fig. 6d). Vitronectin peaked at about 80–100 s and remained associated with the injury site after 200 s. Platelet accumulation was similar in the presence of either the anti-vitronectin antibody or an isotype-matched control antibody conjugated to Dylight 488 (Fig. 6b,e).

To determine whether vitronectin was bound to platelets, the endothelium or both, we blocked platelet accumulation with eptifibatide^26^. In the absence of platelet thrombus formation after vascular injury, vitronectin accumulation was not significantly reduced (Fig. 6c,d) and the time course of the median integrated fluorescence associated with vitronectin in eptifibatide-treated mice parallels that of the untreated mice (Fig. 6d). These results suggest that vitronectin accumulates at the site of injury on the endothelial surface. Furthermore, vitronectin accumulation in the absence of platelet thrombus formation suggests that plasma-derived vitronectin and not platelet-derived vitronectin is the primary substrate of PDI and that PDI released from the endothelium is sufficient to reduce vitronectin at the site of injury.

To appreciate the architecture of the thrombus and its relationship with vitronectin, we performed three dimensional confocal intravital imaging. Confocal stacks were acquired before vessel wall injury and after vessel wall injury at 10 s and at 60 s. PECAM-1, a marker for the endothelium, was imaged with anti-CD31 conjugated to Dylight 550, vitronectin with a mouse monoclonal antibody to mouse vitronectin conjugated to Dylight 488, and platelets with anti-CD42b conjugated to Dylight 649 (Fig. 6f). At 0 time, the vessel wall was visualised but there was no evidence of either bound platelets or vitronectin. At 10 s, vitronectin is observed on the vessel wall in the absence of platelets. At peak thrombus size at 60 s, a platelet thrombus formed but there was no evidence of significant direct vitronectin-platelet interaction, even on rotational analysis of the three dimensional image.

### PDI inhibition blocks vitronectin accumulation *in vivo*

**Figure 7:**
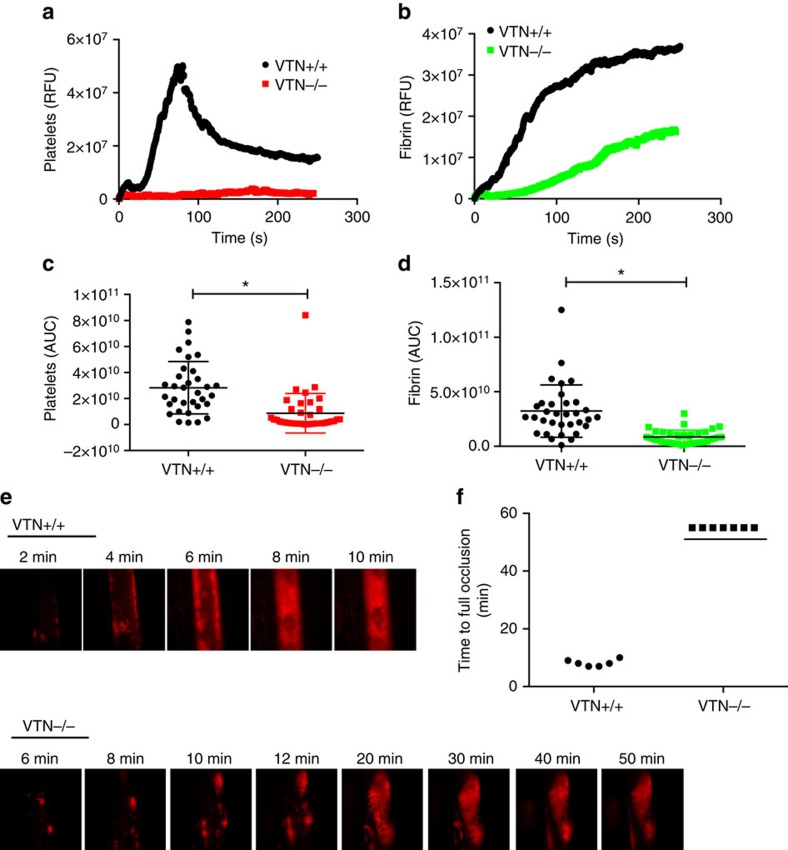
Vitronectin null mice have defective thrombus formation. (**a**,**b**) Vitronectin null mice have reduced platelet accumulation (**a**) and fibrin deposition (**b**) after laser injury in the cremaster arterioles, as visualised by Dylight 649 labelled anti-CD42b and Alexa-488 labelled 59D8 antibody, respectively. The medium fluorescent intensity over time from at least 30 thrombi in each group is shown. (**c**,**d**) Statistical analysis of area under the curve (AUC) for platelets (**c**) and fibrin (**d**) from all thrombi collected in each group is shown. Asterisk indicates statistical significance on Mann Whitney test. (**e**,**f**) Vitronectin null mice have defective thrombus formation induced by FeCl_3_ in the carotid artery. (**e**) Representative images of platelet accumulation (red) in the carotid artery, as visualised by Dylight 649 labelled anti-CD42b, are shown. (**f**) The time to complete vessel occlusion from at least 6 mice in each group was monitored after applying 6% FeCl_3_ on the carotid artery for 1 min^40^.

The function of vitronectin in thrombus formation has been elusive, and analysis of vitronectin null mice^19^, which are viable, has been controversial^18^,^19^. Our hypothesis is that PDI activates vitronectin by cleavage of disulfide bonds, the activated vitronectin can then bind to vessel wall and support thrombus formation. To determine in our model whether vitronectin is required for thrombus formation, we studied thrombus formation in the laser model in vitronectin null mice. No platelet thrombus formation was observed in vitronectin null compared with the wildtype littermates (Fig. 7a,c). Similarly, fibrin generation was significantly diminished (Fig. 7b,d).

To confirm these observations in a separate model of thrombosis, we evaluated thrombus formation in the carotid artery using ferric chloride to initiate vessel wall damage. Wildtype mice showed vessel occlusion by 10 min (Fig. 7e) but vitronectin null mice showed no complete occlusion after 40 min (Fig. 7e). The analysis of the mice studied is presented in Fig. 7f.

**Figure 8:**
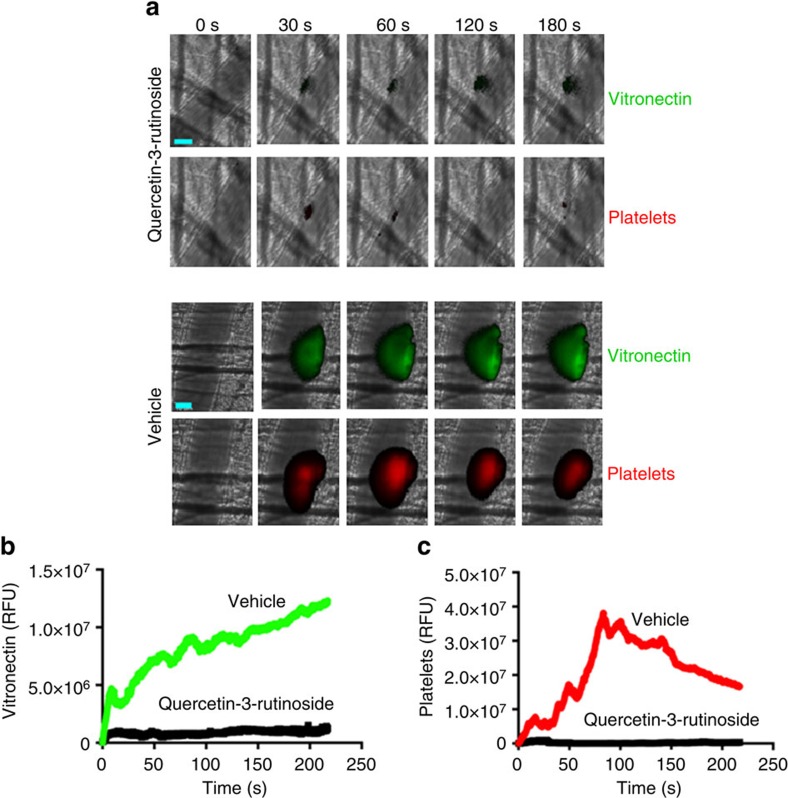
Inhibition of PDI by quercetin-3-rutinoside blocks vitronectin accumulation on the endothelium following vascular injury. (**a**) Infusion of quercetin-3-rutinoside (0.5 μg g^−1^ body weight) 5 min before laser injury (upper panel) or vehicle (lower panel). The kinetics of the median integrated fluorescence of vitronectin (**b**) and platelets (**c**) at the injury site plotted versus time for at least 28 thrombi in 3–4 mice mice. Scale bar (cyan): 20 μm.

Since the absence of vitronectin might have pleiotropic effects, we studied a small molecular inhibitor that inhibits only PDI among the thiol isomerase tested. Quercetin-3-rutinoside is an inhibitor of extracellular PDI in mouse models of thrombosis. Among thiol isomerases, quercetin-3-rutinoside is a selective inhibitor of PDI and does not inhibit ERp5, ERp57, ERp72, thioredoxin or thioredoxin reductase^27^. Given that PDI reduces disulfide bonds in plasma vitronectin that allows vitronectin binding to β_3_ integrins, we explored whether vitronectin becomes associated with the endothelium after vascular injury if PDI is inhibited with quercetin-3-rutinoside. Platelet accumulation is completely blocked by inhibitors of PDI^2^,^27^ so we focused this experiment on the evaluation of vitronectin binding to the endothelium. We have previously demonstrated that sufficient PDI is released from the endothelium at the site of injury to induce vitronectin binding (Fig. 6c,d). Consistent with previous observations^27^, quercetin-3-rutinoside blocked platelet thrombus formation (Fig. 8a,c). However, PDI inhibition by quercetin-3-rutinoside resulted in complete blockade of vitronectin adhesion at the site of injury (Fig. 8a,b). These results implicate PDI in enabling vitronectin binding to the endothelium.

**Figure 9:**
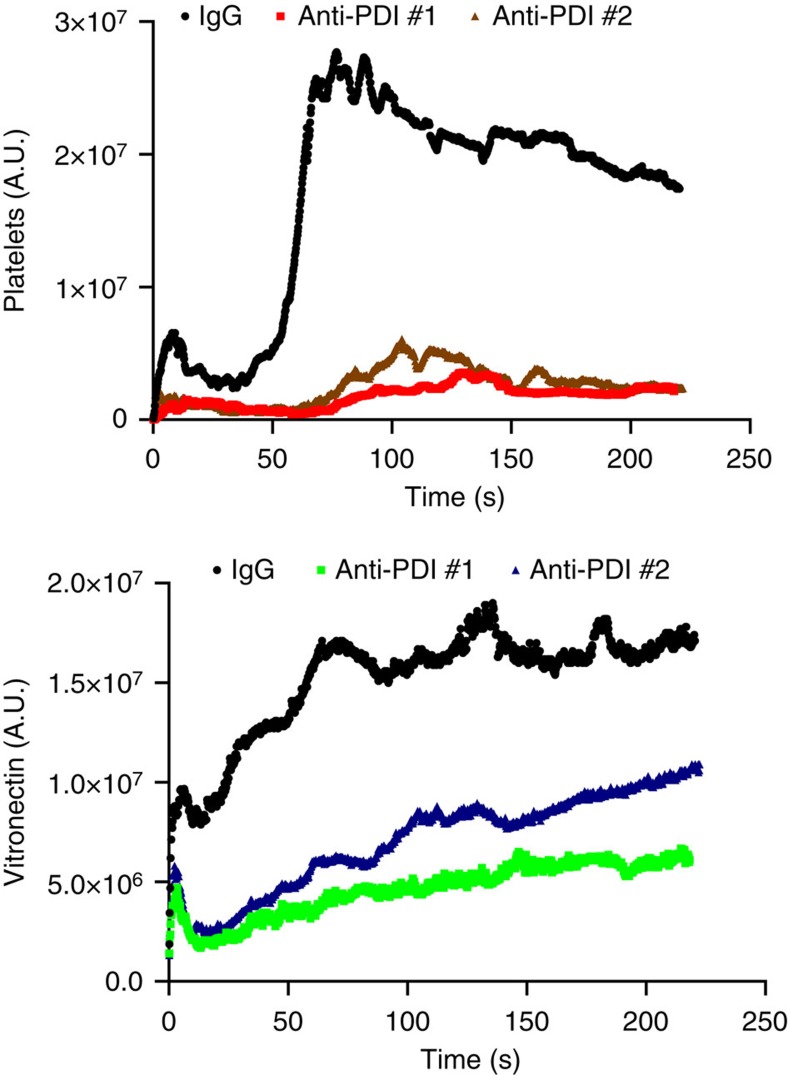
Inhibitory antibodies to PDI block vitronectin accumulation in the developing thrombus. Specific antibodies to PDI, ARP48150 (anti-PDI #1, N-terminal epitope) and H-160 (anti-PDI #2, *b*′-domain epitope), demonstated inhibition of vitronectin accumulation on the vessel wall after infusion of the antibodies (3 μg g^−1^ body weight). The kinetics of the median integrated fluorescence of platelets (upper panel) and vitronectin (lower panel) at the injury site are plotted versus time from at least 30 thrombi in 3–4 mice.

Quercetin3-rutinoside has many other targets. To prove that inhibition of PDI specifically decreases the function of vitronectin in thrombus formation, we used two inhibitory antibodies to PDI that do not cross-react with other vascular thiol isomerases. These antibodies show no binding to ER57, ERp5 or ERp72 (Supplementary Fig. 1). In a functional assay these antibodies show no ability to inhibit function except in the case of PDI (Supplementary Fig. 2). Both anti-PDI antibodies, one against the N-terminal and the other against the b' domain, showed the inhibition of platelet accumulation and significant decrease in vitronectin accumulation (Fig. 9). These results indicate that *in vivo* inhibition of PDI impacts the role of vitronectin in thrombus formation.

## Discussion

Thrombus formation is required to maintain the integrity of a closed, high pressure circulatory system following vascular damage. Vessel wall injury and the extravasation of blood from the circulation rapidly initiate events that seal the breach. Under normal circumstances in the absence of injury, processes associated with thrombus formation remain quiescent in order to maintain patent blood vessels. The molecular and cellular mechanisms necessary to suppress thrombus formation have been thought to depend primarily on spatial sequestration of tissue factor and collagen. However, based on the observation that vascular thiol isomerases, secreted from the activated endothelium and bound platelets following vessel wall injury, are required for platelet thrombus formation and fibrin generation^2^,^3^,^7^,^9^, we now introduce the concept that enzymatic redox modulation of the structures of proteins involved in thrombus formation is important in regulating the initiation of the haemostatic process. This enzymatic process is also controlled by spatial sequestration, in this case of thiol isomerases.

PDI is essential for thrombus formation *in vivo*^2^,^4^. Neither platelet accumulation nor fibrin generation occurs in the injured vascular bed in the absence of the secretion of PDI. A defect in granule secretion in Hermansky–Pudlak syndrome that impairs PDI release contributes to the bleeding disorder characteristic of this syndrome^28^. The mechanism by which the inhibition of PDI eliminates platelet accumulation and fibrin generation is unknown. The function of PDI has been primarily studied within the context of its intracellular role in protein synthesis and disulfide bond formation in the endoplasmic reticulum^29^,^30^,^31^. We have modified a method employed to identify intracellular substrates of thiol isomerases^32^ and identified candidate PDI substrates by mass spectrometry. The candidate substrates include vitronectin, prothrombin, complement factor 3, α2-macroglobulin, histidine-rich glycoprotein, protein S, complement factor 5 and thrombospondin-1.

In the current work, we focused on vitronectin since it is involved in thrombosis^18^ but the molecular basis of its involvement has not been elucidated. The primary pool of vitronectin resides in plasma, where the concentration is about 300 μg ml^−1^ (ref. 33). A platelet pool, stored in the α-granule and released upon platelet activation, contains ∼0.8% of the circulating pool of vitronectin^34^,^35^,^36^. Vitronectin is present *in vivo* in monomeric and multimeric forms, depending on its association with other molecular species and the activity of proteolytic enzymes^37^. PDI catalyses the formation of vitronectin-thrombin-antithrombin III complex *in vitro*^38^, promoting deposition of the ternary complex into the extracellular matrix as well as its clearance from the circulation^39^. However, we did not observe any evidence of vitronectin complexes in plasma before vascular injury. *In vitro* studies examining vitronectin-PDI interaction have used purified protein preparations. Isolation of plasma vitronectin may result in alteration of its native tertiary and quaternary structure as well as its redox potential, leading to changes in binding properties.

Given our observation that plasma vitronectin is a substrate for PDI and that PDI reduces disulfide bonds in vitronectin, the question remains: How does this change in the covalent structure modify vitronectin function? Plasma vitronectin does not bind to the vitronectin receptor, αVβ3 (ref. 17). We hypothesize that upon vascular injury and the secretion of PDI from the endothelium, the intradomain disulfide bonds Cys 137–Cys 161 and the interdomain Cys 274–Cys 543 within the hemopexin domains of vitronectin are reduced, enabling vitronectin binding to αVβ3 and α_IIb_β3 to support platelet aggregation. We do not know currently if one or both of these disulfide bonds need to be reduced in order to enable vitronectin receptor binding. As shown using the ferric chloride model and the laser injury model using vitronectin-null mice, vitronectin supports both platelet thrombus formation and fibrin generation^18^, and our work confirms this In the absence of vitronectin^18^,^19^,^40^ or, as in our studies, in the presence of an inactive plasma vitronectin, thrombus formation is compromised. Using the laser-induced cremaster thrombosis model^41^, Reheman *et al*. demonstrate a 70% reduction in the platelet thrombus and a 50% reduction in fibrin generation^18^ in vitronectin null mice. We showed similar results. Nonetheless, while it is well appreciated that vitronectin plays a role in thrombus formation, how vitronectin functions in this process is not understood. In the absence of platelets, vitronectin collects on the endothelial surface via an unknown mechanism. It remains unclear how vitronectin becomes deposited to the endothelium and how vitronectin may support adhesion under arteriole shear rates. Vitronectin binds not only to αVβ3 and α_IIb_β3, but also the urokinase receptor and PAI-1, via its N-terminal SMB domain^42^,^43^. The intradomain disulfide bond Cys137–Cys161 and the interdomain disulfide bond Cys 274–Cys 543 are located in the hemopexin domains. In the absence of an x-ray structure of full length vitronectin, the specific function of the hemopexin modules in vitronectin are not known. These modules function in other proteins as protein binding modules, and thus may play a role in multidomain interactions. Although we observed PDI-mediated reduction of these two disulfide bonds in the hemopexin domains, this leads to changes in the somatomedin B domain since there is *de novo* exposure of the RGD site important for integrin recognition.

Under normal physiologic conditions thrombus formation is limited to the area of injury and thrombi must not propagate systemically. Three regulatory mechanisms are known to play a critical role in limiting thrombus formation after vascular injury: Tissue factor pathway inhibitor inactivates the extrinsic pathway and thrombin generation is amplified and propagated by the intrinsic pathway^44^,^45^,^46^; the protein C system inactivates the active forms of factor VIII and factor V, turning off thrombin generation^47^,^48^; antithrombin scavenges and inactivates blood clotting enzymes downstream of the area of vascular injury^49^. These regulatory systems limit thrombin activity at the site of injury and act to modulate thrombus formation after initiation of the process has occurred. What regulates the initiation of thrombus formation—that is, What keeps the process of thrombus formation within the intact circulatory system quiescent under normal conditions, thus preventing pathologic thrombosis is unknown. We suggest that the absence of extravascular thiol isomerases in the circulation suppresses thrombus formation. On vascular injury the suppression of thrombus formation is relieved by the local release of thiol isomerases from the injured endothelium and bound platelets. As presented in this first example of the mechanism by which PDI activates downstream components, plasma vitronectin is a substrate for PDI and its functional disulfide bonds are reduced to enable it to bind to its receptors on the endothelium and bound platelets, the β3 integrins, αVβ3 and α_IIb_β3. The reduction of these disulfide bonds expose the RGD sequence that is important for receptor recognition. Once bound, vitronectin can participate in the process of thrombus formation. We hypothesize that PDI specifically and the vascular thiol isomerases more generally covalently modify additional protein substrates involved in thrombus formation, converting them from inactive to active participants, via modification of disulfide bonds^11^. The requirement for thiol isomerase-catalysed modification of proteins important to initiation of thrombus formation is a regulatory switch by which the normal vasculature is protected under physiologic conditions from generating thrombi. Only upon vascular injury do these thiol isomerases, interacting with their array of substrates, allow for initiation of thrombus formation by known agonists? In contrast to previously recognized regulatory mechanisms that modulate thrombus propagation after thrombus formation is initiated, the thiol isomerase system serves as a regulator of the initiation of thrombus formation.

## Methods

### Purification and activity assay of PDI constructs

PDI-CACC (C56A substitution), PDI–CCCA (C400A substitution), PDI–CACA (C56A and C400A substitutions), and PDI–AAAA (C53A, C56A, C397A, C400A substitutions) were constructed using the Phusion Site-Directed Mutagenesis kit (Thermo Scientific) from full-length human PDI cDNA in the pcDNA3 vector provided by Drs Thomas Rapoport and Sol Schulman, Harvard Medical School. The coding sequence for PDI-CCCC (wild type) and the PDI variants was amplified using primers 5′-GCATTGAAGCTTGACGCCCCCGAGGAG-3′ and 5′-CGATACAGATCTCAGTTCATCTTTCACAGCTTTCTG-3′ then transferred to the pT7-FLAG-SBP-1 vector (Sigma) using HindIII and BglII restriction sites. These expression vectors were used to produce PDI fusion proteins containing an N-terminal FLAG epitope (DYKDDDDK) and a C-terminal Streptavidin Binding Peptide. Similar strategies were used to prepare variants of ERp57. PDI and ERp57 proteins were expressed in *E. coli* BL21(DE3)T1 cells (Sigma), purified by streptavidin affinity chromatography (Pierce Biotechnology) followed by dialysis into PBS, pH 7.4. The enzymatic activities of the PDI constructs were evaluated using the insulin reductase assay^50^.

### *Ex vivo* kinetic trapping and 2D gel electrophoresis

Platelet-rich plasma (5 ml) was recalcified to 1 mM CaCl_2_; Gly-Pro-Arg-Pro-NH_2_ (GPRP; 5 mM) was added to inhibit fibrin polymerization during platelet activation. Recombinant PDI or its variants (500 μg) were incubated with 20 mM DTT at 0° for 20 min, desalted then added to recalcified platelet-rich plasma followed by addition of collagen (10 μg ml^−1^) and human α-thrombin (0.2 U ml^−1^). The samples were incubated for 3 min at 23° before adding hirudin (20 U ml^−1^) then 20 mM N-ethylmaleimide (NEM). The supernatant containing plasma and platelet releasate was isolated by centrifugation. PDI, variants and their disulfide-linked complexes were precipitated using streptavidin-agarose beads. The streptavidin beads were thrice washed with 25 mM Tris (pH 6.8), 500 mM NaCl, 1.5% Triton X-100 and 20 mM NEM. Bound PDI and complexes were eluted with 3 mM biotin. Proteins were analysed by SDS–PAGE on 3–8% gradient gels (Life Technologies) under non-reducing and reducing conditions and transferred onto nitrocellulose membranes (Bio-Rad).

Preparative gel electrophoresis was performed with non-reduced samples run in the first dimension on SDS–PAGE using 7% gels. The gel lane was excised, incubated in SDS sample buffer with 2% DTT, followed with 2.5% NEM for 20 min in the dark at 23°. The gel strip was positioned on another 7% SDS–PAGE gel. Following separation, proteins were visualised by silver staining. Protein spots of interest were identified containing the trapping variant PDI-CCCA by comparison with those of PDI-CCCC. Selected protein spots were subjected to in-gel trypsin digestion and the peptides analysed by LC/MS/MS.

### Western blot analysis

Samples from trapping experiments were analysed by immunofluorescence Western blot analysis under non-reducing and reducing conditions. PDI and vitronectin were probed simultaneously using 1:1000 dilution of mouse anti-DYKDDDDK (clone 9A3, Cell Signalling) against the FLAG tag on PDI and 1:1000 dilution of rabbit anti-human vitronectin anti-serum (Complement Technologies), then visualised by 0.2 μg/ml of goat anti- mouse Alexa 488 and goat anti-rabbit Alexa 647 (Life Technologies), respectively. Dual-labelled immunofluorescent images were obtained using the ImageQuant LAS4000 (GE Healthcare). Full scans of Western blots are presented in Supplementary Fig. 3.

### Immunocytochemistry

Cells were stained for DAPI and vitronectin using rabbit anti human vitronectin (1:1000 dilution, Complement Technologies of anti-serum) and goat-anti rabbit labeled with Alexa 488 (10 μg/ml), and imaged by fluorescence microscopy.

### Dieosin disulfide reductase assay

Dieosin glutathione disulfide, Di-Eo-GSSG, was synthesized by the reaction of eosin isothiocyanate with GSSG. Di-E-GSSG had low fluorescence which increased upon reduction of its disulfide bond^51^. Reductase activity of purified enzymes was monitored in 96-well fluorescence microtiter plates. Recombinant ERp5, ERp72, PDI and ERp57 were assayed at the concentrations indicated in the absence or presence of anti-PDI antibody directed at an epitope in the b′ domain (H-160; Santa Cruz) or at an N-terminal epitope in PDI (PH4B; Aviva) (3 μM). Di-Eo-GSSG (150 nM) was added to enzyme in the presence of 5 μM DTT and the increase in fluorescence due to release of eosin-glutathione for ERp5, ERp72, ER57 and PDI was determined by excitation at 520 nm and emission at 545 nm in a Synergy 4 plate reader,Winooski VT). The reduction of 150 nM di-E-GSSG by 5 μM DTT alone served as a negative control.

### PDI-mediated disulfide bond reduction in vitronectin

The free thiols of platelet-poor plasma proteins were alkylated by the addition of 50 mM NEM for 20 min at 23° in the dark. After dialysis, the NEM-pretreated plasma was recalcified to 1 mM CaCl_2_. PDI–CCCC, PDI–AAAA or ERp57–CCCC as indicated was added to 100 μl of NEM-pretreated plasma in 0.3 mM DTT. The reaction was monitored and the reaction terminated by alkylation of new thiols using 20 mM of PEG_5000_-maleimide (Sigma-Aldrich) or PEG_2000_-maleimide (Nanocs). Aliquots were examined by SDS–PAGE and probed for vitronectin by Western blotting. The PDI-mediated reduction of vitronectin was monitored by changes in the electrophorectic mobility due to alkylation with PEG_2000_-maleimide or PEG_5000_-maleimide.

### Identification of the PDI-cleaved disulfide bond(s)

Trapping experiments were performed with PDI–CACA as above except that platelet-free plasma was used. The reaction was stopped with 50 mM NEM. PDI and PDI complexes were isolated with streptavidin beads and eluted with biotin. Agarose-TCEP reduced the PDI–S–S–X complexes and 50 mM iodoacetamide (IAM) alkylated newly formed thiols. After reduction and alkylation vitronectin was immunoprecipitated using anti-human vitronectin antibodies conjugated to coupled to protein G Dynabeads (Invitrogen), the protein purified by affinity chromatography, resolved by SDS–PAGE, then stained with Coomassie blue. The vitronectin band was excised from the gel, digested with trypsin, and the modified cysteines identified using an Orbitrap Velos Pro (Thermo Scientific). Peptide sequences were determined from the fragmentation pattern using Sequest.

### Solid-phase binding of vitronectin to β3 integrins

α_V_β_3_ integrin (EMD Millipore) or αIIbβ3 (Molecular Innovations) was applied to microtiter wells (250 ng per well) overnight, then blocked with BSA. PDI–CCCC, PDI–AAAA, ERp57-CCCC or BSA were added with 0.3 mM DTT to 100 μl of recalcified plasma. The reaction proceeded for 10 min at 37 °C and terminated with 20 mM NEM. Plasma was diluted 1:100, added to the coated plates for 1 h, and bound vitronectin quantitated with rabbit anti-vitronectin antibody detected with anti-rabbit IgG conjugated to HRP.

### Adhesion of PDI-reduced plasma vitronectin to HUVECs

HUVECs (Lonza, Catalogue # C2519A) were grown on collagen type I–coated coverslips (BD Biosciences) in EGM-2 media (Lonza). Serum-starved washed cells were activated with human α-thrombin (1 U ml^−1^) and 1 mM MnCl_2_ for 10 min at 37 °C. After washing, thrombin was inhibited with hirudin (5 U ml^−1^). Cells were treated with GRGESP (5 μM), GRGDSP (5 μM) or an anti-α_V_β_3_ monoclonal antibody (clone LM609 (10 μg ml^−1^; EMD Millipore) for 30 min. PDI-CCCC (15 μM) or PDI-AAAA (15 μM) was added to recalcified plasma (100 μl) containing 5 μM GPRP and 0.3 mM DTT. The reaction was incubated at 37 °C for 5 min, diluted 1:10 with EGM-2 medium containing 1 mM MnCl_2_ then added to the activated HUVECs. After 30 min, the cells were washed, fixed with 4% paraformaldehyde then blocked with 10% goat serum. Cells were stained for DAPI and vitronectin using rabbit anti human vitronectin and goat-anti rabbit labelled with Alexa 488, and imaged by fluorescence microscopy.

### Mice

The Beth Israel Deaconess Medical Center Institutional Animal Care and Use Committee approved all animal care and experimental procedures. All mice were male and were 8 to 12 weeks old. Wild type mice were obtained from Jackson Laboratories (Bar Harbor ME). Vitronectin-null mice were rederived by Jackson Laboratories from mice prepared and deposited by Ginsburg and colleagues^40^.

### Intravital microscopy of laser-induced arteriolar thrombosis

Intravital microscopy of the cremaster microcirculation was performed using the laser-injury model^41^. Platelets were labelled with CD42b conjugated to Dylight 649 and vitronectin labelled with monoclonal anti-mouse vitronectin or isotype IgG conjugated to Dylight 488. In some experiments, eptifibatide^3^,^26^ or anti-PDI antibodies or quercetin-3-rutinoside^27^ was infused before injury. Image analyses were comprised of data sets of >28 thrombi in 3–4 mice per experiment^27^ using Slidebook v5.5 (Intelligent Imaging Innovations).

### Carotid artery thrombosis assay

Ferric chloride induced carotid artery thrombosis assay was performed as an independent monitor of the role of vitronectin in thrombus formation^52^,^53^. Mice were anaesthetised by intraperitoneal injection of a mixture of ketamine and xylazine and a cannula tube was prepared in the left jugular vein for the administration of Nembutal (to maintain anaesthesia) and antibodies^41^. The right common carotid artery was then exposed and platelets were labelled by infusion of Dylight 649 conjugated anti-CD42b antibody (0.1 μg g^−1^ body weight)(Emfret Analytics). Thrombus formation was initiated by applying Whatman filter paper (1 mm by 3 mm) pre-soaked in ferric chloride (6%) solution to the carotid artery for 1 min. The carotid artery was then washed and superfused with pre-warmed (37 °C) and aerated (5% CO_2_, 95% N_2_) bicarbonate buffered saline^41^. Platelet accumulation and thrombus formation was monitored under an intravital fluorescent microscope and the time to complete blood flow occlusion was recorded. In all cases, the time to complete vessel occlusion was determined only after the vessel had remained closed for 20 min.

### Data availability

All relevant data and the supporting materials are available from the corresponding author on request.

## Additional information

**How to cite this article:** Bowley, S. R. *et al*. Protein disulfide isomerase secretion following vascular injury initiates a regulatory pathway for thrombus formation. *Nat. Commun.* **8,** 14151 doi: 10.1038/ncomms14151 (2017).

**Publisher's note:** Springer Nature remains neutral with regard to jurisdictional claims in published maps and institutional affiliations.

## Supplementary Material

Supplementary Information Supplementary Figures.
