# Supplementary material for: Protein disulfide isomerase secretion following vascular injury initiates a regulatory pathway for thrombus formation
Source: Nat Commun. 2017 Feb 20;8:14151. doi: 10.1038/ncomms14151 (PMC5321760; doi:10.1038/ncomms14151)
Supplement: Supplementary Information — Supplementary Figures. [file ncomms14151-s1.pdf]

Supplementary Data

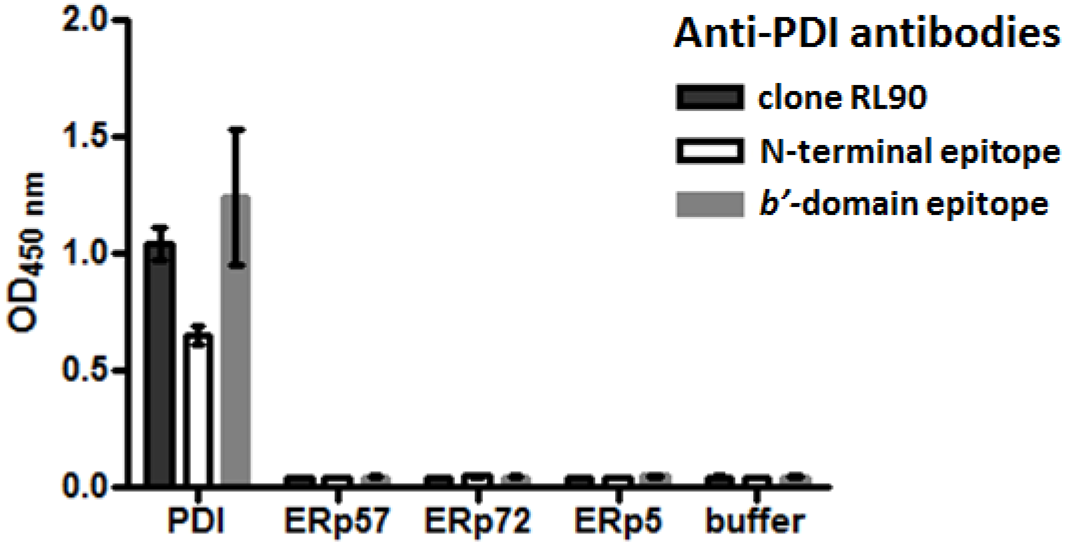

Supplementary Fig. 1. Anti-PDI antibodies to bind to PDI and not other vascular thiol isomerases. Rabbit polyclonal antibodies to PDI targeting the N-terminal amino acid residues 100-150 (ARP48150, Aviva Systems Biology) and the b' region amino acid residues 211-370 (H-160, Santa Cruz Biotechnology) were tested for cross-reactivity to other thiol isomerases by enzyme-linked immunosorbent assay (ELISA). Either PDI, ERp57, ERp72 or ERp5 was coated onto microtiter wells (250 ng/well) overnight and then blocked with BSA. The two rabbit polyclonal antibodies, as well as a monoclonal antibody to PDI (RL90, Novus Biologicals) were individually applied to each thiol isomerase coated well and binding was detected using anti-rabbit or anti mouse IgG conjugated to horseradish peroxidase HRP followed by reaction with the chromogenic substrate tetramethylbenzidine at 450 nm. Anti-PDI: RL90, black; ARP48150, white; H-160, gray.

A

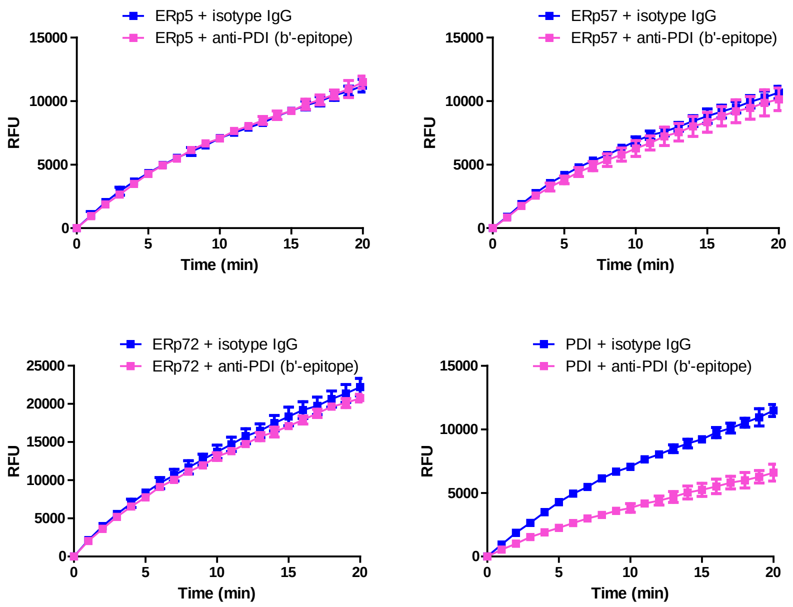
ERp5 + isotype IgG
ERp5 + anti-PDI (b'-epitope)
RFU
0
5000
10000
15000
Time (min)
0
5
10
15
20
ERp57 + isotype IgG
ERp57 + anti-PDI (b'-epitope)
RFU
Time (min)
ERp72 + isotype IgG
ERp72 + anti-PDI (b'-epitope)
RFU
0
5000
10000
15000
20000
25000
Time (min)
PDI + isotype IgG
PDI + anti-PDI (b'-epitope)
RFU
Time (min)

B

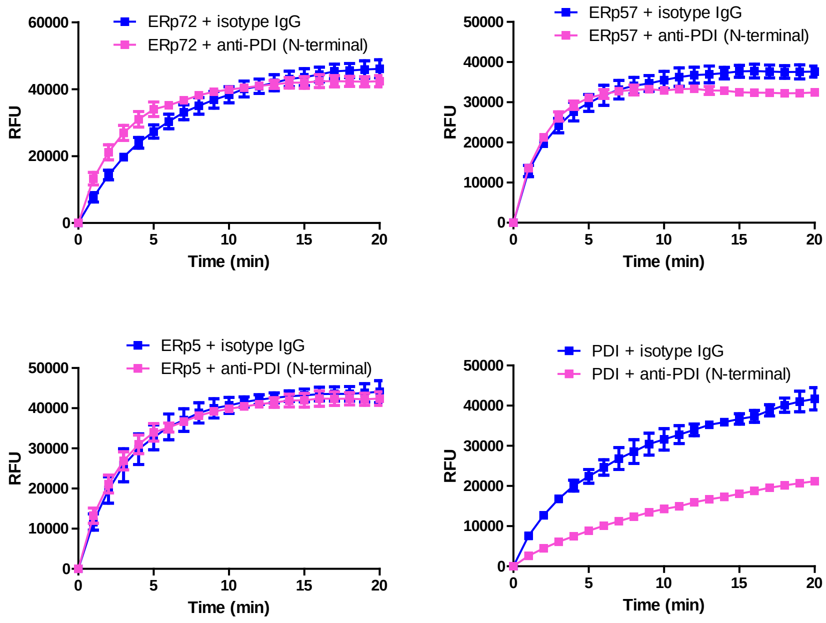
ERp72 + isotype IgG
ERp72 + anti-PDI (N-terminal)
RFU
0
20000
40000
60000
Time (min)
0
5
10
15
20
ERp57 + isotype IgG
ERp57 + anti-PDI (N-terminal)
RFU
0
10000
20000
30000
40000
50000
Time (min)
ERp5 + isotype IgG
ERp5 + anti-PDI (N-terminal)
RFU
Time (min)
PDI + isotype IgG
PDI + anti-PDI (N-terminal)
RFU
Time (min)

Supplementary Fig. 2. Specificity of antibodies to PDI and their crossreactivity with other vascular thiol isomerases. To determine whether PDI is required for vitronectin activation, antibodies to PDI were examined in the Di-Eo-GSSG functional thiol isomerase assay using independently the H-160 and the PH4B polyclonal antibodies against different domains of PDI. A. H-160 antibodies against the b' domain of PDI; B. ARP48150 directed against the N polyclonal antibodies against PDI. No inhibition of functional activity of PDI was observed with the addition of vascular isomerase other than PDI.

Supplementary Figure 3. Western blots, uncropped. The images in Fig. 2 were cropped, labeled and composited. The original tiff files are shown below, A and BC, correspond to Fig. 2ABC

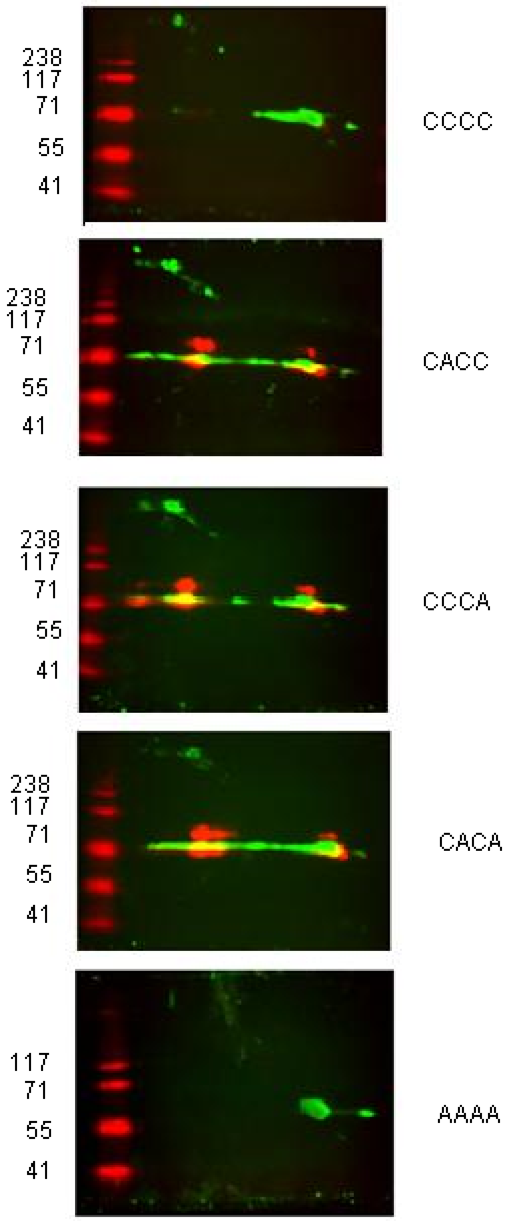

Fig.2A

Fig. 2BC

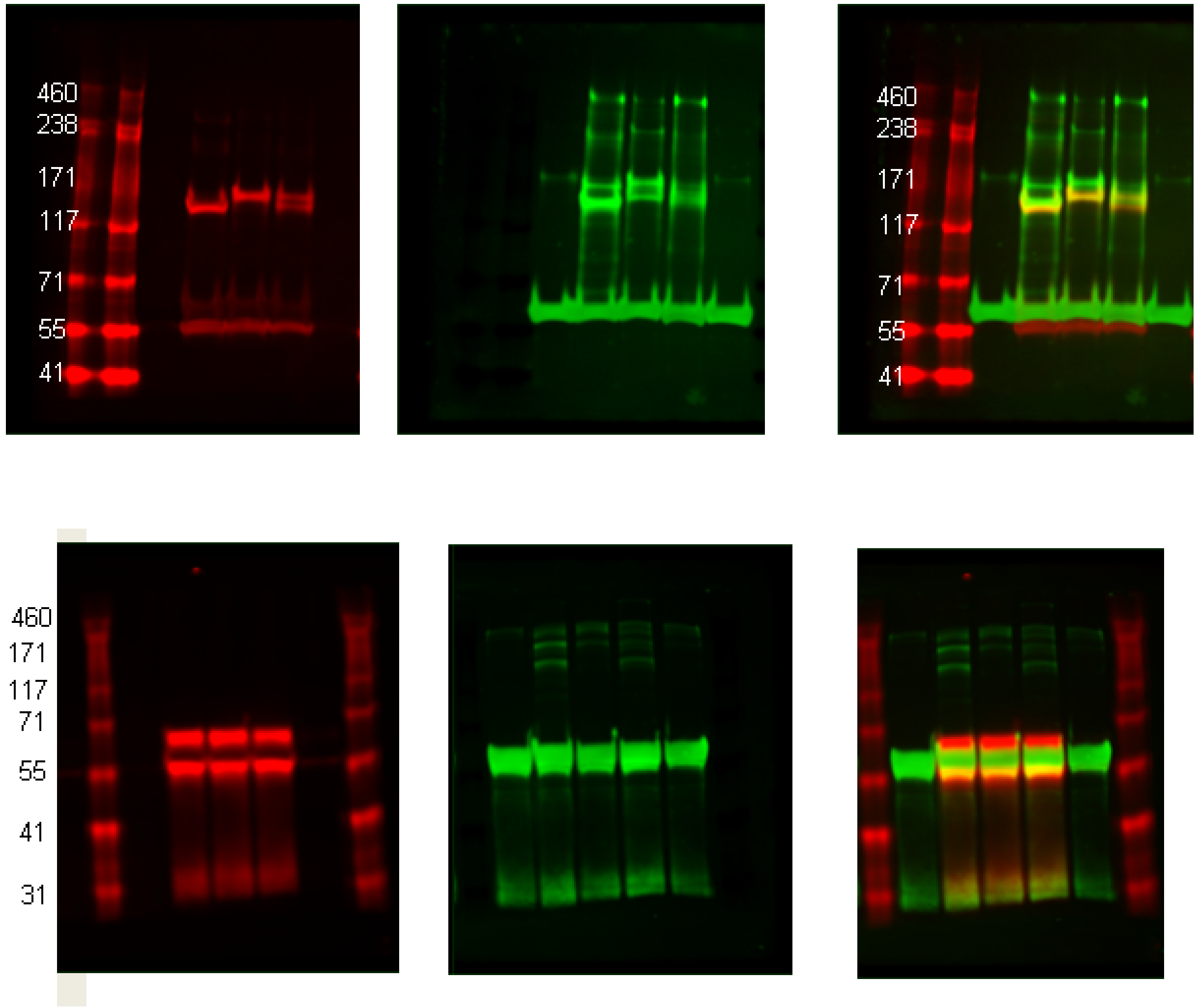
460
238
171
117
71
55
41
460
238
171
117
71
55
41
460
171
117
71
55
41
31
